# β-Thalassemia Minor and Pregnancy Outcomes: Pathophysiology, Clinical Implications, and Management

**DOI:** 10.3390/medsci14020225

**Published:** 2026-04-30

**Authors:** Angeliki Gerede, Sofoklis Stavros, Efthymios Oikonomou, Anastasios Potiris, Maria Danavasi, Vasiliki Kourti, Ismini Anagnostaki, Ekaterini Domali, Nikoletta Koutlaki, Makarios Eleftheriadis

**Affiliations:** 1 Department of Obstetrics and Gynecology, Democritus University of Thrace, 69100 Alexandroupolis, Greece; efthymiosoikonomou@gmail.com (E.O.); mairidanavasi@gmail.com (M.D.); vasiliki.kourti29@gmail.com (V.K.); nkoutlak@med.duth.gr (N.K.); 2 Third Department of Obstetrics and Gynecology, University General Hospital “ATTIKON”, Medical School, National and Kapodistrian University of Athens, 12462 Athens, Greece; sfstavrou@med.uoa.gr (S.S.); apotiris@med.uoa.gr (A.P.); 3 Medical School, National and Kapodistrian University of Athens, 11528 Athens, Greece; isanagnostaki3@gmail.com; 4 First Department of Obstetrics and Gynecology, Alexandra Hospital, Medical School, National and Kapodistrian University of Athens, 11528 Athens, Greece; kdomali@yahoo.fr; 5 Second Department of Obstetrics and Gynecology, “Aretaieion” University Hospital, Medical School, National and Kapodistrian University of Athens, 11528 Athens, Greece; melefth@med.uoa.gr

**Keywords:** β-thalassemia minor, pregnancy outcomes, maternal anemia, fetal growth restriction, postpartum hemorrhage, iron supplementation, transfusion management

## Abstract

β-thalassemia minor, often referred to as the β-thalassemia trait, is among the most prevalent hemoglobinopathies globally, impacting around 80–90 million carriers, with a prevalence of up to 15% among Mediterranean, Middle Eastern, and Asian populations. Although traditionally regarded as clinically benign, pregnancy imposes hematologic and metabolic stressors that may unmask latent vulnerabilities. This review combines the latest data and findings about the pathophysiology of β-thalassemia minor during pregnancy, its short-term outcomes on the mother and fetus, and its long-term impact on the child, as well as management techniques. A narrative review of PubMed-indexed studies (2000–2025) was conducted, including cohort and case–control studies, systematic reviews, meta-analyses, and international guidelines. Outcomes were organized by theme, and quantitative findings (prevalence, relative risks, odds ratios) were combined when available. Anemia is a common health issue for mothers. Literature mentions that the pooled incidence is between 30% and 40% during the third trimester, with ~5%of carriers needing a blood transfusion (mainly in iron-deficient or baseline Hb 6–8 g/dL cases). Meta-analyses have shown elevated risks of pre-eclampsia (odds ratio (OR) ~ 1.4, 95% confidence interval (CI) 1.1–1.8) and postpartum hemorrhage (PPH); however, estimates differ by region. The odds of preterm delivery (OR ~ 1.4), small-for-gestational-age (SGA) (OR ~ 1.5), and low birth weight (LBW) are slightly increased for carriers, and neonatal intensive care unit (NICU) admission rates are also higher for carriers. However, the risk of stillbirth is not always increased. The usual approach is iron supplementation guided by ferritin levels to prevent overload, personalized transfusion thresholds, and regular folate support. There is not much evidence for long-term consequences for children of carrier mothers since no research has followed more than 200 children born to carrier mothers into adulthood. However, maternal anemia is linked to slower growth, neurodevelopmental issues, and a higher risk of cardiometabolic problems in larger groups of pregnant women. However, maternal anemia is associated with slower growth, neurodevelopment, and higher cardiometabolic risk in larger groups of pregnant women. β-thalassemia minor during pregnancy usually has a mild, though significant, impact. While most pregnancies proceed without complications, this condition is associated with a significantly higher prevalence of anemia and other adverse postnatal outcomes. Consequently, the implementation of risk-stratified monitoring, smart supplementation, and standardized management protocols is essential. Prospective registries, mechanistic placental research, and long-term offspring cohorts are necessary to better understand long-term trends.

## 1. Introduction

β-thalassemia is a set of inherited hemoglobinopathies that occurs when the body does not produce enough or any β-globin chains of hemoglobin. This causes microcytosis and different levels of anemia [1]. The heterozygous condition, referred to as β-thalassemia minor or trait, is generally regarded as clinically silent or linked solely to modest hematological irregularities, including borderline anemia and microcytosis [2]. Pregnancy may be a physiological stressor that reveals or exacerbates an underlying hematological imbalance, which raises questions regarding the hazards to the mother and fetus.

Epidemiologically, β-thalassemia is very common in the Mediterranean region, the Middle East, South and Southeast Asia, and certain areas of Africa [2,3]. In regions like Cyprus, Sardinia, and Southeast Asia, carrier frequencies may reach 10–15%. This is mostly due to evolutionary selection pressures against malaria [4]. β-thalassemia is now a major health issue in pregnant women around the world because global migration has spread it beyond its usual endemic areas [3]. There are an estimated 80 to 90 million carriers around the world. Even minor risks in pregnancy outcomes could have a significant impact on public health [3].

In antenatal populations, reported carrier rates are similar to the background rates in the area: multiethnic clinics in Europe and North America report a carrier frequency of ~1–5%, while programs across the Mediterranean and Middle East report a carrier frequency of ~5–12%, and several South/Southeast Asian programs report a carrier frequency of ~10–15% [3,4]. In endemic countries, prevalence is variable—often heightened in coastal or historically malarial areas—underscoring the need for tailored screening and counseling strategies [2,3].

Due to the classification of β-thalassemia minor as “benign” by physicians, there has been less research on its implications during pregnancy. However, there is an increasing number of studies that report possible associations with maternal anemia, increased transfusion demands, and negative obstetric outcomes such as preterm birth, LBW, and increased demand for neonatal care [5]. The above-mentioned risks may be influenced by nuanced pathophysiological mechanisms such as relative placental hypoxia and the maternal hematopoietic system’s failure to accommodate a highly demanding gestation. Importantly, there is still no agreement on how to handle the situation, especially when it comes to iron supplements and the requirement for transfusion support from time to time.

The goal of this review is to compile a complete and focused summary of the existing evidence on β-thalassemia minor during pregnancy. We will (a) present the pathophysiological mechanisms and their impact on the mother and fetus, (b) report the outcomes of pregnancy for both the mother and child, (c) summarize clinical management strategies, and (d) study the available data on the long-term hematological outcomes in children born to carrier mothers. Doing so, we hope to fill up the existing gaps in our understanding and set priorities for future research.

## 2. Materials and Methods

This narrative review was based on a structured literature search conducted in PubMed. Search terms included both MeSH headings and free-text keywords (title/abstract), such as “β-thalassemia minor,” “thalassemia trait,” “pregnancy,” “maternal outcomes,” “fetal outcomes,” “obstetric complications,” “anemia,” “low birth weight,” “preterm birth,” and “management.” Boolean operators were used to combine thematic concepts and refine retrieval.

The search included only peer-reviewed, English-language articles published mainly in the past six years (January 2019 and March 2026) to emphasize contemporary clinical practices and recent meta-analyses. It should be noted that earlier high-impact studies from 2000 onwards were included to provide epidemiological or mechanistic context. This review combined original research articles, cohort and case–control studies, systematic reviews, meta-analyses, and clinical guidelines from professional bodies (Royal College of Obstetricians and Gynecologists—RCOG, American College of Obstetricians and Gynecologists—ACOG, World Health Organization—WHO).

Articles were screened and selected independently by the author based on title, abstract, and relevance to the research objectives. Studies were thematically categorized under four main domains: (a) pathophysiology and maternal–fetal implications, (b) pregnancy outcomes, (c) management and guideline-based recommendations, and (d) long-term outcomes for the offspring.

The gathered data and findings were synthesized narratively to highlight epidemiological patterns, summarize clinical outcomes, and detect potential knowledge gaps. Quantitative findings such as prevalence, relative risks, and ORs were incorporated where available to provide a clearer picture of the maternal and neonatal burden.

## 3. Pathophysiology and Maternal–Fetal Implications

The main cause of β-thalassemia minor is heterozygous mutations in the β-globin gene, which lead to a reduced synthesis of β chains and an imbalanced ratio of globin chains [6]. All those extra unpaired α chains pile up, which messes with red blood cell production. Although many people who carry β-thalassemia minor do not develop symptoms until later in life, the accumulation of excess alpha chains impairs red blood cell formation and results in microcytic, hypochromic red blood cells and mild, chronic anemia [7]. The body’s natural increase in blood volume and increased demand for red blood cells during pregnancy may exacerbate these abnormalities and potentially lead to anemia.

The World Health Organization (WHO) defines anemia in pregnancy as a hemoglobin (Hb) level of <11 g/dL in the first and third trimesters and <10.5 g/dL in the second trimester [8]. Studies have shown that pregnant women with the β-thalassemia trait typically have hemoglobin levels of 10–11 g/dL, while controls have 12–13 g/dL. Microcytosis, indicated by an MCV around 65 fL, and decreased MCH values are also typical [9]. While women with β-thalassemia minor often maintain a baseline Hb of 10–11 g/dL, there is still disagreement among clinicians about whether these conventional thresholds are right for this group of people adjusted for baseline microcytosis (MCV ~ 65 fL) [10]. Given their reduced baseline, a ‘physiologic’ drop in Hb due to gestational hemodilution may lead to earlier classification of anemia compared to non-carriers, while it should be noted that, even in such cases, women may not always exhibit the functional phenotype of iron-deficiency anemia [11]. Relative literature estimates that up to 40% of reproductive-age women in endemic areas have concurrent iron deficiency, increasing transfusion risk if untreated [12,13]. While trait carriers show mildly suppressed hepcidin and elevated soluble transferrin receptor (sTfR) due to ineffective erythropoiesis, oral iron (30–60 mg elemental/day if ferritin is <30–100 μg/L + CRP adjustment) poses negligible overload risk with no cases reported in pregnancy cohorts. Withholding iron heightens maternal/fetal risks (OR 1.8 for SGA) [14,15]. Anemia is exacerbated by the fact that the red cells in individuals with β-thalassemia minor contain less hemoglobin per unit of volume [6]. Therefore, the increased blood volume and demand for oxygen experienced during pregnancy further reduce the oxygen-carrying capacity of the mother’s blood, making it even more difficult for the mother to provide sufficient oxygen to the developing fetus [16]. This subclinical placental hypoxia could explain the higher rates of adverse obstetric outcomes, such as preterm birth and fetal growth restriction in carriers. Recent meta-analyses suggest that between 65% and 80% of pregnant carriers experience anemia, a figure that contrasts with the approximate 30% prevalence observed in the broader obstetric population; furthermore, 5% to 10% may require transfusions at some stage during their pregnancy [17,18]. These estimates were obtained from larger thalassemia populations and include limited data regarding the minor phenotype. Other disorders may affect diagnosis and treatment options for some populations and should be considered in planning diagnostic and therapeutic approaches to individuals with β-thalassemia minor [7].

Further complicating the issue of pathophysiology is iron metabolism. The management of iron metabolism in β-thalassemia minor during pregnancy requires a structured approach that balances the risk of deficiency against the theoretical risk of overload. Iron deficiency is common in women of reproductive age; however, concerns have been raised regarding iron overload in carriers who take nondiscriminate supplementation. In β-thalassemia, the body’s red cell production is so inefficient that it suppresses hepcidin (the hormone that controls iron absorption), potentially leading to iron overload and changes throughout the body—even in the carrier state [19,20]. Clinical studies have indicated that up to 45% of β-thalassemia minor carriers exhibit at least one marker of renal tubular dysfunction, including microalbuminuria or any abnormal potassium/uric acid excretion, representing chronic erythroid stress and low-level hypoxic injury [9]. Ineffective erythropoiesis in β-thalassemia minor mildly suppresses hepcidin through modest erythroferrone elevation, preserving the hepcidin/ferritin axis with only small changes (normal/low hepcidin/ferritin ratio), but overload from oral iron is undocumented in pregnancy [21,22]. Studies show no iron overload from oral supplements in trait carriers and no toxicity [23,24]. In fact, membrane defect cohorts exhibited high levels of sTfR but normalized with iron correction without excess accumulation while hepcidin remained responsive (unlike in cases of major thalassemia) [23,24]. Thus, overload risk from pregnancy dosing (30–60 mg/day) is negligible, prioritizing deficiency treatment. Because withholding iron for a theoretical risk of overload may exacerbate maternal anemia and increase the subsequent need for blood transfusions, supplementation should be guided by ferritin levels rather than avoided entirely. Moreover, differences in study design and methodology have resulted in variable findings across studies, as illustrated by the disparate findings of Ruangvutilert et al. [25] and Thilakarathne et al. [14], indicating that regional or methodological differences may account for discrepancies. Furthermore, while chronic, low-grade inflammation and oxidative damage to red blood cells in carriers may indirectly impair placental function and elevate the likelihood of adverse fetal development and preterm birth in pregnancies [19], ensuring adequate iron stores is vital to maintaining maternal oxygen-carrying capacity. A substantial amount of the currently available data came from cohorts that include nonpregnant populations or populations with more severe clinical manifestations, highlighting the clear need for well-designed research specifically focusing on the relationship between iron homeostasis and pregnancy outcomes in the β-thalassemia minor population.

Concerning the fetus, if the mother has anemia and placental hypoxia, its intrauterine growth can slow down, sometimes leading to LBW or being SGA. β-thalassemia minor itself does not pass along the blood deficiency to the fetus—unless both parents are carriers—but the indirect effects from the mother’s anemia are still important. Pooled data from over 8000 pregnancies show carriers have a 1.6 times higher risk of preterm birth and a 1.5 times higher risk of intrauterine growth restriction compared to women without the trait, though results vary a lot between studies [17]. Epidemiologic research consistently reports that carriers face a 1.5–2 times greater risk of intrauterine growth problems and early delivery than non-carriers [17]. Therefore, even though most of what we know comes from indirect evidence, there is a solid biological reason for the links between β-thalassemia minor, anemia, and poorer pregnancy outcomes seen in these studies.

## 4. Pregnancy Outcomes in β-Thalassemia Minor

Although β-thalassemia minor is generally regarded as a benign carrier state, pregnancy may bring out problems that might otherwise stay hidden. Evidence from observational studies and meta-analyses in thalassemia populations highlights increased risks for both mothers and infants, even when disease severity is milder than in transfusion-dependent phenotypes.

### 4.1. Maternal Outcomes

The most consistent maternal complication in β-thalassemia minor is anemia. As a result of normal hemodilution that occurs in pregnancy and the increased baseline microcytosis associated with this condition, many patients will have hemoglobin levels well below what would be considered “normal” or relative cut-off points. While the overall prevalence of anemia in carriers rises to 30–40% by the third trimester, carriers are almost twice as likely as non-carriers to have Hb < 10 g/dL [17]. However, the clinical requirement for transfusion remains a topic for discussion among clinicians. Some patients experience symptomatic anemia necessitating red cell transfusions [18]; however, reported transfusion rates vary widely based on regional factors. For instance, in a case–control study from Sri Lanka, 18.3% and 16.7% of carriers required transfusion in the second and third trimesters, respectively [14]. It is important to note that such high rates are often observed in cohorts where baseline Hb is exceptionally low (6–8 g/dL), likely due to co-existing nutritional deficiencies rather than the carrier state alone. In Western cohorts, transfusions are rarely required for the “otherwise healthy” carrier. Misclassification with iron deficiency remains a challenge, especially in regions with high rates of nutritional deficiencies, making both diagnosis and treatment difficult [17]. It is very crucial to make an accurate diagnosis since untreated iron deficiency, not the thalassemic trait itself, is frequently the primary driver of severe anemia that requires a transfusion.

Reviews and cohort studies also indicate that carriers of beta-thalassemia minor are at a greater risk of various complications of pregnancy, including an increased frequency of obstetric bleeding, including postpartum hemorrhage (PPH), and more frequent cases of hypertension in pregnancy, specifically pre-eclampsia [17,18], as well as an increased likelihood of cesarean deliveries [26]. In a study of 1288 women from Thailand with thalassemia traits (including 424 with β-thalassemia minor) compared with 1305 controls, pregnancy-induced hypertension occurred in 6.9% versus 4.7% (*p* = 0.018), with the β-thalassemia trait having a relative risk of 1.67 (95% CI: 1.11–2.51) [25]. However, not all studies agree with these findings: while some studies reported significantly increased rates of hypertensive disorders [25], other studies have failed to demonstrate an increased risk of hypertension in pregnancy in beta-thalassemia minor carriers [14], which indicates that several factors, such as ethnicity, nutritional status, and availability of medical care, influence the results obtained in the studies. A meta-analysis by Falcone et al. (2022) reported a pooled OR for pre-eclampsia of 1.4 (95% CI: 1.1–1.8), but heterogeneity across studies was high (I^2^ > 50%), reflecting inconsistent definitions and variable adjustment for confounders [17]. The reason for such associations could be the mechanisms of endothelial dysfunction and oxidative stress, as well as micronutrient imbalances that accompany chronic anemia and ineffective erythropoiesis [27].

Pregnancy might cause a physiological hypercoagulable state, which in turn can increase the risk of venous thromboembolism (VTE) by 4–5 times via venous stasis, endothelial alterations, and elevated levels of various clotting factors [28]. The β-thalassemia trait further exacerbates this condition by chronic RBC membrane instability, phosphatidylserine exposure, platelet hyperreactivity, and endothelial microparticle release, establishing the conditions for the development of a prothrombotic environment even in non-splenectomized carriers [29]. Unstable microcytic RBCs are characterized by 3–5 times faster phosphatidylserine externalization, which activates tissue factor pathways, while low-grade hemolysis depletes nitric oxide, affecting, in this way, the process of vasodilation [30]. Chronic ineffective erythropoiesis elevates platelet activation markers such as P-selectin [30]. Meta-analyses demonstrate a VTE incidence of 1.7–9.2% among thalassemias, which is 10 times higher than the general population; however, data on minor traits are limited in the current literature [31]. Currently, there are no extended pregnancy-specific cohorts focusing on trait carriers, but thrombosis rates during pregnancy for thalassemia intermedia are 3 times higher than those of thalassemia major (0.92%), suggesting a dose-response with disease severity [18]. Splenectomized patients are at the highest risk (OR 2.8) due to thrombocytosis, while non-splenectomized ones are at a lesser risk (~1–2%) but still significant in pregnancy [32]. All β-thalassemia trait pregnancies receive low-dose aspirin (75 mg/day) from 12 weeks. Splenectomized patients with platelets >600 × 10^9^/L require combined low molecular weight heparin (LMWH) plus aspirin prophylaxis. High-risk cases, such as those with a history of VTE, immobility, or multiple gestation, warrant prophylactic LMWH regardless of splenectomy status.

In general, the cardiovascular and metabolic risks associated with beta-thalassemia minor are not as great as those associated with the more severe forms of beta-thalassemia, but its impact should not be underestimated, especially in settings where a precise diagnosis or an urgent transfusion is not always possible. Extended obstetric cohorts with women with thalassemia major confirm the high severity of complications in severe phenotypes [33], so it is important to proceed with caution even when evaluating the minor form. Nevertheless, Ruangvutilert et al. found that almost one third of the carriers were anemic in the third trimester compared to just 12% of controls (*p* < 0.001), highlighting that hematologic malfunction is both common and clinically relevant [25]. Taken together, cross-study comparisons suggest that although gestational anemia is always more common in carriers, the severity of adverse complications such as postpartum bleeding or high blood pressure varies geographically and demographically, including parameters such as genetics, nutrition, and healthcare infrastructures.

### 4.2. Fetal and Neonatal Outcomes

There are several important fetal/neonatal risks to both the mother and the infant associated with the condition. The limited availability of oxygen in the placenta, along with maternal anemia, can lead to serious implications for the developing fetus. There are documented increased risks for preterm births, LBW, SGA babies, decreased amniotic fluid levels (oligohydramnios), and miscarriages when pregnant compared to nonpregnant controls [17,18,26]. A recent study found that women who had anemia during their first trimester had nearly two times greater odds of having a child that was born SGA (adjusted OR 1.89; 95% CI: 1.35–2.64) and were at an increased risk of LBW and/or requiring hospitalization in the NICU.

Stillbirth and neonatal mortality are not consistently increased in carriers; however, there is very little literature available regarding this issue, and most outcomes will depend upon the maternal co-morbidities and the quality of healthcare available to the mother [34]. The results of the Sri Lanka case–control study indicated that LBW occurred in approximately 17.1% of carriers vs. 15% of controls (*p* = 0.80), and NICU admissions were essentially the same as well [14]. The meta-analysis by Falcone et al. indicates that the pooled ORs of premature birth among carriers are ~1.4 (95% CI: 1.1–1.8) and ~1.5 (95% CI: 1.2–1.9) for small-for-gestational age; however, these authors also noted heterogeneity within the estimates, which can be attributed to differences in methodologies and variability in the true prevalence of the effects within different populations [17]. However, these findings indicate that regional variations can occur significantly based on maternal nutritional status, maternal iron levels, and quality of maternal healthcare.

For example, Ruangvutilert et al. found a significant association with small-for-gestational age [25], while Thilakarathne et al. did not and therefore suggest that maternal nutritional status, maternal iron status, and quality of maternal health care could influence the risk of adverse neonatal outcomes [14]. Furthermore, additional factors such as iron deficiency, folate deficiency, or hyperhomocysteinemia may contribute to an increased risk of adverse neonatal outcomes, but these have not been thoroughly examined in individuals with the minor phenotype [27]. In summary, when examining the available literature, the increased risk of adverse neonatal outcomes is generally not high but consistent. Estimates typically fall between 1.3 and 1.8 for preterm birth, SGA, and LBW. Therefore, β-thalassemia minor is not a direct cause of neonatal morbidity but does create conditions for vulnerability.

Population-based data from a study from Israel further support increased fetal risks while reporting oligohydramnios and IUGR (OR 2.1 and 2.4, respectively) among 261 β-thalassemia minor pregnancies, alongside higher cesarean rates (16.9%), though perinatal mortality remained comparable [35]. Similarly, a study from Iran found significantly elevated oligohydramnios (10.8%) and cesarean deliveries (38.3%) in 510 I thalassemia minor pregnancies, but no differences in preterm birth, LBW, or pre-eclampsia [36]. These findings underscore amniotic fluid and growth surveillance needs despite overall favorable outcomes.

Table 1 summarizes the main reported maternal and fetal outcomes in pregnancies with β-thalassemia minor.

## 5. Management During Pregnancy

Beta-thalassemia minor is best managed by a personalized approach during pregnancy through a combination of routine obstetrical care and blood-related issues. Many women have no symptoms, and most will remain undiagnosed; however, a demanding pregnancy has the potential to make clinically significant anemia apparent and may increase the risk of adverse outcomes related to delivery.

### 5.1. Monitoring

Monitoring is essential for patient management and includes regular measurements of hemoglobin, hematocrit, and serum ferritin to determine whether the woman has iron deficiency or is a thalassemic carrier [37]. It must be noted that ferritin is an acute-phase reactant, and therefore, serum inflammatory markers such as CRP may need to be evaluated in conjunction with ferritin measurements. Fetal growth assessments using serial ultrasound may be required to evaluate for intrauterine growth restriction (IUGR), especially in women who remain anemic during their pregnancies. In one case series of pregnant thalassemic women, approximately 40% of them required additional fetal growth assessment because their hemoglobin levels remained below 10 g/dL, despite iron supplementation [10].

### 5.2. Iron Supplementation

Iron supplementation is significantly different from the general obstetrical population. The offspring of a β-thalassemia minor carrier mother have a 50% chance of inheriting the trait (normal hematology), and 50% are normal. If the father is also a carrier, there is a 25% chance of major, 50% trait, and 25% normal—warranting counseling/prenatal testing [38]. Routine iron supplementation is not presumptively provided for women carrying β-thalassemia minor because excessive iron accumulation can occur extensively in β-thalassemia carriers due to uncontrolled ineffective erythropoiesis and uncontrolled regulation of hepcidin [39]. Hepcidin suppression is minimal (e.g., 1.2–1.5× erythroferrone compared to controls), insufficient for overload at standard doses, as ferritin rose appropriately (not excessively) in supplemented traits [23,24]. Therefore, iron supplementation should be guided by serum ferritin levels, and replacement should be done when serum ferritin levels are less than 30 µg/L (<100 µg/L with concomitant inflammation) according to WHO and ACOG. Oral formulations should be used as the first-line treatment; however, if oral treatments fail or cause intolerable side effects, then intravenous formulations may be used in the second or third trimester. There are recent reports indicating that as high as 25–30% of β-thalassemia carriers receive indiscriminate iron supplementation, which may lead to increased risk of iron overload after delivery if not carefully monitored and controlled [10,14].

### 5.3. Transfusions

Transfusions are rarely needed in β-thalassemia minor; however, they may be indicated in women with severe anemia that is resistant to iron therapy or in situations of obstetric PPH. The threshold for transfusion should be determined individually; however, transfusions should be reserved for hemoglobin levels <7 g/dL or symptomatic maternal compromise. Transfusions should not be liberal, as they carry a risk of alloimmunization and iron overload. Evidence from case studies indicates that ~5% of women with compound thalassemia traits or severe anemia episodes may require at least one transfusion during pregnancy, with a target hemoglobin level ≥10 g/dL to minimize maternal and fetal complications [10].

### 5.4. Guideline-Based Recommendations

Current recommendations from RCOG, ACOG, and WHO utilize a risk-based approach: universal screening for anemia in pregnancy, appropriate iron replacement based on biochemical parameters, and transfusions only for severe or symptomatic cases. Additionally, it is recommended that all women with hemoglobinopathies receive folic acid supplementation (5 mg/day) to support erythropoiesis and prevent the development of neural tube defects. The Thalassemia International Federation also emphasized preconceptual counseling and individualized monitoring, stating that proactive management of beta-thalassemia major carriers during pregnancy could decrease the rate of obstetric complications by up to 20% compared to unscreened populations.

**Figure 1:**
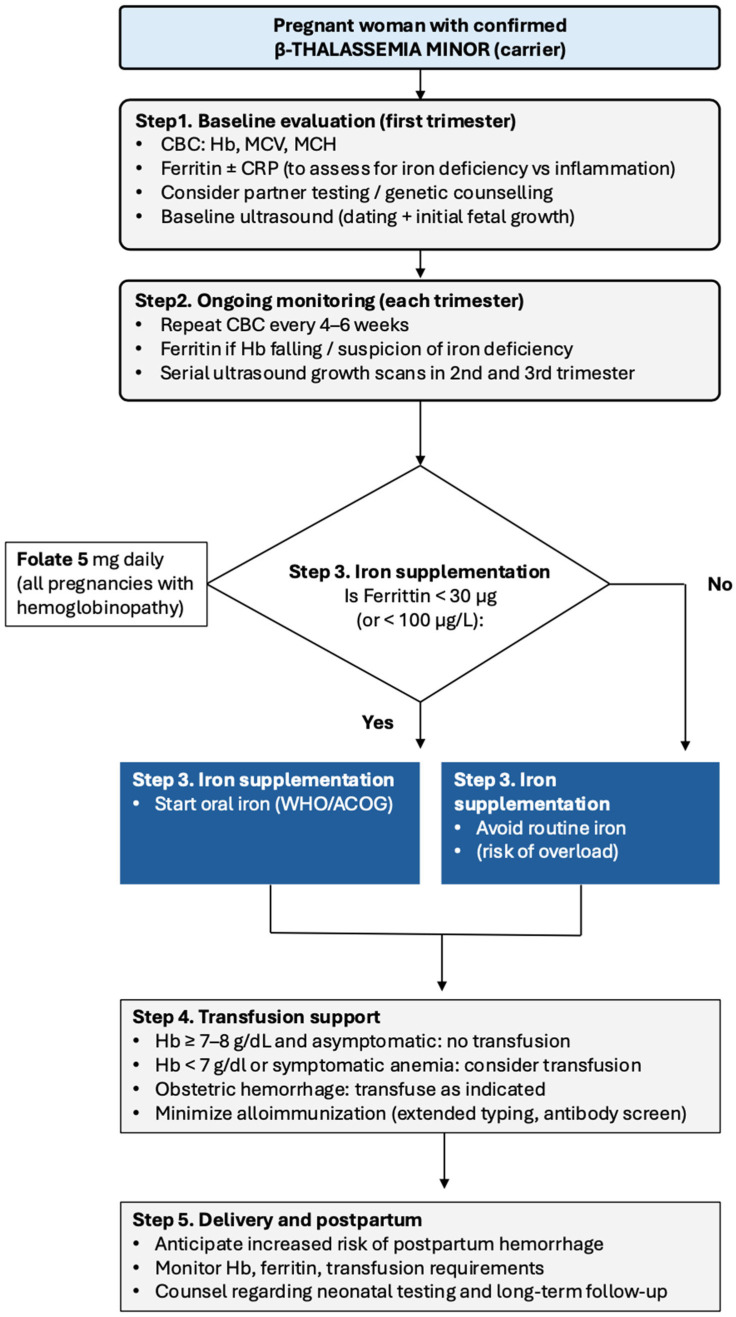
Suggested management algorithm for β-thalassemia minor in pregnancy.

In summary, these strategies support early identification and individualized management of women with beta-thalassemia minor, rather than routine interventions. Figure 1 outlines a systematic method for managing beta-thalassemia minor during pregnancy that includes monitoring, iron supplementation, and transfusion assistance in accordance with current evidence-based guidelines. Visual summaries such as algorithms standardize the care of women with beta-thalassemia minor and identify areas for further research, including the long-term balance between iron deficiency and iron overload in this population.

## 6. Long-Term Outcomes for Offspring

There exists limited research regarding the long-term effects on children of mothers who have β-thalassemia minor. Most of the existing information is extrapolated from studies on β-thalassemia major or intermedia, which have several chronic complications related to their condition (e.g., chronic anemia, transfusion burden, and iron overload), which can lead to serious implications for the child’s growth, endocrine system, and heart [40]. Traditionally, it has been assumed that individuals who carry the gene for β-thalassemia are clinically asymptomatic, and therefore, there has been little interest in monitoring the health of their children over time.

In terms of a hematologic standpoint, newborns born to mothers who are carriers of β-thalassemia typically will not exhibit clinical manifestations unless both parents are carriers. In such cases, the offspring would be at risk of developing β-thalassemia major, and therefore genetic counseling and prenatal testing would be warranted. However, if only the mother is a carrier of the gene, the offspring will likely have normal hematologic values at birth, but few longitudinal studies exist to confirm the persistence of these findings through late childhood [41]. A population-based retrospective cohort study of singletons born between 1991 and 2014 demonstrated that children whose mothers carried the gene for β-thalassemia minor had significantly more frequent hematologic hospitalizations (3.3% vs. 0.7%, *p* < 0.001), and this association persisted after adjusting for maternal age, SGA status, gestational age, and birth weight, with maternal β-thalassemia minor being independently associated with long-term hematologic morbidity in their offspring (adjusted hazard ratios of 5.54, 5.56, and 5.49 (with 95% confidence intervals (CI) ranging from 3.63 to 8.44, 3.65 to 8.47, and 3.60 to 8.36, respectively) and all statistically significant with *p* < 0.001) [26].

Beyond the aspect of genetics, there may be other indirect maternal influences that impact the health and well-being of their offspring. Maternal anemia during pregnancy has been correlated with decreased fetal growth, lower iron levels in the fetus at birth, and greater risk of delayed development in the broader obstetric population [42]. For instance, a large cohort analysis of over 15,000 pairs of mothers and children demonstrated that maternal anemia increases the ORs of low neonatal iron status by 1.8 times and is correlated with a 25% higher risk of delayed psychomotor development at 2 years of age [43]. Additionally, a 2022 population-based study conducted in China reported that children born to anemic mothers had a 1.3-fold higher risk of being stunted or underweight during early childhood [44]. Longitudinal follow-up studies also indicate that maternal anemia (of whatever cause) may predispose children to poor academic achievement, reduced cognitive function, and greater numbers of behavioral problems [44]. These conclusions are largely based on research from non-thalassemia populations, and therefore, it has not been clearly established whether these risks are specific to children of mothers who are β-thalassemia carriers, since most prior studies did not distinguish between the effects of having β-thalassemia minor and those of having iron-deficiency anemia.

Recent studies provide evidence that there may be potential cardiometabolic consequences to the offspring of mothers who experience maternal anemia in utero. Studies have shown that the previously mentioned offspring are at a higher risk of developing hypertension, insulin resistance, and abnormal lipid profiles during adolescence [43,44]. While these studies provide indirect evidence that the subtle hypoxia produced by β-thalassemia minor may influence cardiometabolic programming, very few studies have investigated this issue using cohorts.

Thus, the current evidence base shows a clear research gap. There are a small number of studies that have provided some systematic follow-up data on children born to mothers who are β-thalassemia carriers; however, these studies have relatively small sample sizes (less than 200), and none of the studies have followed more than 200 offspring of carrier mothers into adulthood, nor have they systematically evaluated outcomes beyond the age of 18 years [44]. Furthermore, no large-scale, prospective longitudinal studies have systematically evaluated the hematologic, developmental, and/or metabolic outcomes of the offspring of β-thalassemia carriers. Therefore, given the high frequency of the β-thalassemia carrier state among people from the Mediterranean, Middle East, and Asia regions, there is a significant unmet need for maternal-child health research to investigate whether the carrier state produces unique risks for the offspring of carrier mothers. Future studies should focus on distinguishing the contributions of β-thalassemia minor from co-existing nutritional deficiencies and assessing whether small reductions in placental oxygenation produce significant risks for the offspring.

## 7. Discussion

β-Thalassemia minor has long been considered a benign condition, as it is generally asymptomatic. However, there have recently been findings suggesting that β-thalassemia minor can cause pregnancy complications due to several possible mechanisms. One such complication is maternal anemia, which is present in a higher number of pregnancies in β-thalassemia minor carriers compared to non-carriers. Maternal anemia may need to be differentiated from iron deficiency, since some studies indicate that a considerable proportion of the studies included in this review have misclassified anemic carriers as being deficient in iron [17,25]. There have also been heterogeneous definitions of anemia used throughout the studies, different baseline populations studied, and no consistency in how potential confounding factors were controlled. These concerns result in considerable variation in reported maternal and fetal risks related to β-thalassemia minor carriers. Consequently, much of the evidence provided in this review should be considered suggestive of potential risks rather than conclusive, necessitating a cautious approach. Contrary to severe thalassemias, trait carriers exhibit hepcidin responsiveness, which minimizes overload from oral iron, as demonstrated by stable iron indices following supplementation [2,23,24]. Excessive caution may exacerbate a deficiency common in Asian/Mediterranean pregnancies. In addition, many of the studies reviewed here can identify associations or trends with various adverse pregnancy outcomes; however, many studies do not provide strong enough evidence to conclusively determine if there is a causal relationship between β-thalassemia minor and each of the respective adverse pregnancy outcomes. These issues are at least partially responsible for the observed inconsistency in whether β-thalassemia minor carriers have a higher incidence of premature birth, LBW, or PPH as compared to the general population [14,34]. While meta-analysis supports this variability, the pooled OR for premature birth and SGA in carriers varies from 1.3 to 1.8; however, the variability among regions remains high [17]. Quantitative evidence supports the conclusion that β-thalassemia minor is a moderate, yet consistent, risk factor for adverse pregnancy outcomes; however, the magnitude of this risk factor is significantly enhanced by the presence of nutritional deficiencies and inadequate prenatal care.

Therefore, it is essential to differentiate β-thalassemia minor from thalassemia major and intermediate forms of the disease. Severe forms of the disease, such as thalassemia major and intermediate forms, are associated with significantly higher rates of maternal and fetal complications, such as the necessity for transfusions, cardiovascular complications, and poor perinatal outcomes [33]. Conversely, individuals who are carriers of β-thalassemia minor rarely require transfusions and are only at a slight increase in risk of adverse pregnancy outcomes. It would be incorrect to extrapolate results from major and intermediate forms of the disease to the carrier state, as this could lead to an overestimation of the risk of carriers and would not reflect the clinical nuances within the literature. Therefore, it is essential to explicitly separate the three forms of the disease to avoid overestimating the risk of carriers and to maintain the clinical nuance of the literature.

Although the above-stated limitations exist regarding β-thalassemia minor, some clinical implications emerge. The interaction between chronic microcytic anemia, the dilutional effect of pregnancy on maternal hemoglobin levels, and the need for adequate placental oxygen delivery suggests that women who are carriers of β-thalassemia minor cannot be classified as “low-risk” for pregnancy-related complications. Rather, close monitoring of maternal hemoglobin levels and ferritin levels, as well as individualized supplementation strategies and judicious use of transfusions, are recommended [37,39]. Specifically, fetal growth surveillance should be emphasized, as all the studies reviewed here have demonstrated a consistent association between β-thalassemia minor and SGA infants [17,18]. The proposed management algorithm for pregnant women with β-thalassemia minor is shown in Figure 1. For clinicians, especially those practicing in endemic regions, practical priorities include: (i) confirming carrier status early and providing genetic counseling/testing to their partners; (ii) utilizing ferritin (±CRP) levels to guide iron replacement therapy and avoid empirically treating carriers with iron when ferritin ≥30 µg/L; (iii) increasing surveillance when maternal hemoglobin levels fall <10 g/dL or decline rapidly despite the administration of oral iron; (iv) performing serial ultrasound assessments of fetal growth in the late second and third trimesters; (v) reserving blood transfusions for carriers with symptomatic anemia or significant decreases in maternal hemoglobin levels with clear maternal–fetal indications; and (vi) documenting both maternal hemoglobin levels and ferritin levels postpartum, as well as newborn testing, for future pregnancies [10,14]. An emphasis on risk-based management of β-thalassemia minor carriers is supported by cohort data indicating that approximately 80% of carriers do not require blood transfusions during pregnancy; however, this percentage can approach 33% in resource-poor regions [14,25].

To improve risk assessment and management, there are several priorities: (1) prospective multicenter registries that have standardized criteria to differentiate minor from intermediate/major and specify both maternal and neonatal outcomes; (2) analytic controls to reduce misclassification due to iron deficiency and inflammation (ferritin with CRP and/or hepcidin when available) [19,20]; (3) mechanistic studies of placental biology (angiogenesis, oxygen delivery, oxidative stress) that correlate maternal hematologic profiles with fetal growth [27]; (4) pragmatic clinical trials to compare ferritin-guided vs. routine iron therapy (oral and IV) and to evaluate the effect of transfusions on both maternal and neonatal outcomes [10]; (5) Longitudinal cohort studies of offspring through adolescence to examine hematologic, neurodevelopmental, and cardiometabolic trajectories [43,44]; and (6) Implementation research to integrate thalassemia screening into antenatal anemia programs, including the cost-effectiveness of partner testing as well as regional pathways for countries located within the Mediterranean, Middle East, and Asia [4].

The future research will have several priorities. First, prospective multicenter studies are required to determine whether β-thalassemia minor has an independent negative impact on pregnancy outcome or if it is simply a marker of nutritional deficiencies that exist with this condition. Secondly, studies that investigate the biological mechanisms involved in placental function, oxygen transfer across the placenta, and the adaptive response of the fetus to hypoxia through erythropoiesis will help understand the pathophysiologic relationship between maternal carrier status and fetal growth. Finally, follow-up of the offspring of these women continues to be a critical area of study; the current data do not allow us to make conclusions regarding the long-term hematologic, developmental, and/or cardiometabolic outcomes of the children born to these women. Specifically, the lack of studies that follow more than 200 offspring of carrier women beyond adolescence represents a significant gap in our understanding of the long-term health of the children of these women.

Finally, as gene editing technology and other new treatments for β-hemoglobinopathies [45] continue to develop, the perception of β-thalassemia minor may change, and therefore, the importance of continued surveillance and thoughtful counseling of individuals in reproductive settings is important. Overall, the results of the current literature support the dual message of this review, i.e., while most pregnancies in carriers occur without serious complications, the carrier state is not entirely benign, and both the practice of medicine and research should be based on this reality.

## 8. Conclusions

β-thalassemia minor is typically viewed as a clinically silent condition; however, pregnancy can reveal the underlying vulnerabilities of this condition that result in implications for both the mother and fetus. In general, most pregnancies in carriers occur without major complications; however, the increased risk of maternal anemia and the potential need for blood transfusions, along with the subtle increase in the frequency of adverse neonatal outcomes, suggest that the carrier state is not completely innocuous. Careful monitoring of the hematologic status of the carrier woman, appropriate differentiation from iron-deficient states, and the implementation of targeted individualized supplementation regimens are all components of managing β-thalassemia minor in pregnancy. Overall, β-thalassemia minor in pregnancy is generally mild but not inconsequential and indicates the need for tailored surveillance and further prospective research to clarify long-term outcomes for offspring.

## Figures and Tables

**Table 1:** Summary of reported maternal and fetal outcomes in pregnancies affected by β-thalassemia minor.

Study	Country/Population	Study Design (n)	Maternal Outcomes	Fetal/Neonatal Outcomes	Key Limitations
[17]	Italy, Multicenter	Retrospective cohort (~350 pregnancies)	↑ Maternal anemia; occasional transfusion; ↑ PPH; ↑ pre-eclampsia	↑ Preterm birth; ↑ LBW; ↑ SGA	Single-country, retrospective; iron deficiency not fully excluded
[18]	Greece	Systematic review and cohort synthesis (n > 1000)	↑ Anemia; transfusion needed in subset; ↑ risk of PPH	↑ Preterm delivery; ↑ SGA; ↑ NICU admission	Heterogeneity of included studies; small sample sizes
[14]	Sri Lanka	Prospective case–control	↑ C-section rate (statistically significant, no definite indications) ↑ transfusions (2nd/3rd trimester) due to lower Hb	No significant differences in complications	Small sample, transfusions likely due to co-existing iron deficiency
[27]	Lebanon	Observational case–control	↑ Anemia; endothelial dysfunction markers	Association with SGA and impaired growth	Small sample; mechanistic focus, not outcomes
[36]	Iran	Case–Control	↑ Anemia, ↑ Transfusion, ↑ C-section	↑ Oligohydramnios	Small sample; single-center focus, unclear diagnostic criteria for β-thalassemia minor confirmation
[33]	Canada, hemoglobinopathy registry	Large cohort (mostly major/intermedia; included minor)	Severe outcomes in major/intermedia; minor less affected	Poorer outcomes mainly in severe phenotypes; minor largely benign	Outcomes not disaggregated clearly for minor
[35]	Israel	Retrospective cohort	↑ C-section 16.9%	↑ Oligohydramnios, ↑ IUGR	Retrospective registry data; no iron status assessment; no transfusion data; 1988–2002 era

↑: increased, PPH: postpartum hemorrhage, LBW: low birth weight, SGA: small for gestational age, NICU: neonatal intensive care unit.

## Data Availability

No new data were created or analyzed in this study.

## Abbreviations

The following abbreviations are used in this manuscript:

ACOG	American College of Obstetricians and Gynecologists
CI	Confidence Interval
IUGR	Intrauterine Growth Restriction
LBW	Low Birth Weight
NICU	Neonatal Intensive Care Unit
OR	Odds Ratio
PPH	Postpartum Hemorrhage
RCOG	Royal College of Obstetricians and Gynecologists
SGA	Small-for-Gestational-Age
sTfR	Soluble Transferrin Receptor
VTE	Venous Thromboembolism
WHO	World Health Organization
